# Should We Continue to Tell Autistic People that Their Brains are Different?

**DOI:** 10.1177/00332941231174391

**Published:** 2023-05-05

**Authors:** Daniel Crawshaw

**Affiliations:** School of Psychology, 6123 University of Nottingham, University Park, Nottingham, UK

**Keywords:** Autism, autism spectrum disorder, heterogeneity, validity, neurodiversity, social constructionism, social identity theory

## Abstract

Autism is often considered to reflect categorically ‘different brains’. Neuropsychological research on autism spectrum disorder (ASD) however, has struggled to define this difference, or derive clear-cut boundaries between autism and non-autism. Consequently, restructuring or disbanding the ASD diagnosis is becoming increasingly advocated within research. Nonetheless, autism now exists as a salient social construction, of which ‘difference’ is a key facet. Clinical and educational professionals must influence this cautiously, as changes to autism’s social construction may counterproductively affect the quality of life of autistic people. This paper therefore reviews ASD’s value as both neuropsychological and social constructs. Although lacking neuropsychological validity, the autism label may be beneficial for autistic self-identity, reduction of stigma, and administering support. Whilst a shift away from case-control ASD research is warranted, lay notions of ‘different brains’ may be preserved.

## Introduction

The notion that autistic individuals are categorically different from the typically developing (TD) population is a prevalent one. Autism is often interpreted to be the *cause* of how an autistic person thinks and behaves, with a spectrum of behaviours and abilities considered to be ‘autistic traits’ (Harris, 2016). As such, autism exists as a salient social label (Chapman, 2020), pivotal to the self-identities, social relationships, and support available to many autistic individuals. It is often perceived that autistic individuals are who they are *because* they are autistic, they simply have ‘different brains’ (Botha et al., 2020).

Defining this difference that autistic brains exhibit however, has been particularly challenging. Researchers have struggled to encapsulate autism spectrum disorder as a unitary set of behavioural traits, or demonstrate a clear distinction between autism, typical development, and other neurodevelopmental disorders (Waterhouse & Gillberg, 2014). Furthermore, no causal psychological or neurobiological difference has been agreed upon, as none seem compellingly unique to or universal within autism (Brunsdon & Happé, 2014). Given the immense heterogeneity which exists in ASD, many researchers are now arguing that autism has vague scientific meaning and poor biomedical utility. Some have called, convincingly, for the concept of autism to be disbanded in neurobiological and psychological research (Müller & Amaral, 2017; Waterhouse et al., 2016).

These calls to disband autism however, scarcely discuss how this might translate into real world use of the autism label. Autism now exists as a prominent socially constructed group, independent of its use in biomedical contexts (O’Reilly & Lester, 2017). Its influence is substantial, and it most affects individuals who often experience social difficulties, worsened by unfavourable judgements and stigmatisation towards autism (Han et al., 2022). Consequently, there is an ethical responsibility that the depiction of autism presented by clinical and educational professionals balances scientific accuracy with being genuinely beneficial to autistic individuals. To achieve this, it should promote self-understanding and self-acceptance, combat unfavourable stigma, and facilitate productive support for autistic difficulties. It must also be accessible, as nuanced scientific arguments may be misconstrued by lay audiences, who might wrongly interpret that autistic peoples’ experiences and challenges are not genuine. In this sense, autism’s meaning is fragile, and an attempt to change or disband it as a social label, based on ASD’s poor validity as a neuropsychological group, might be premature.

Nonetheless, the discrepancy between the scientific reality and social construction of autism invites a reconsideration of whether ‘autistic brains are different’ should be promoted or resisted by clinical and educational professionals. To my knowledge, no other scientific literature has thoroughly discussed the social consequences of labelling autistic individuals in the light of autism’s neuropsychological controversy. It should be noted at this stage, that as this review discusses autism’s meaning as both neuropsychological and social constructs, this may unintentionally detach the autism label from autistic people. As this was written by a non-autistic author, and to ensure autistic participation within autism research, the manuscript was reviewed by an autistic early career researcher to ensure that the language and content is appropriate.

This review begins by discussing the two contrasting meanings of ‘autism’. First autism as a biomedical group, which is poorly supported by neuropsychological evidence, then secondly how this is translated and adapted within autism’s social construction. It is highlighted that “different brains” is a key facet of both. In an attempt to link the two fields, this uses language from both traditional experimental neuropsychology (e.g., TD, ASD), and more recent social psychology (e.g., neurotypical, neurodivergent), to provide a fair representation of evidence discussed. Three evaluations of autism’s social construction are then made. It is argued that the label is largely beneficial for the self-identities of many autistic individuals, as it promotes self-understanding and positive group-membership. Furthermore, notions of ‘different brains’ do not inherently elicit stigma, and label disclosure may positively impact social perceptions. Finally, although the label does not inform autism-specific treatment, it may be economically necessary for allocating support. Henceforth, although autism lacks neuropsychological meaning, this review ultimately argues that social constructions of ‘different brains’ should be preserved.

## What Does ‘Autism’ Actually Mean?

### Autism as a biomedical construct

‘Autism’ has assumed a broad range of meanings in the last century, regularly shifting in both its scientific and sociopolitical conceptualisation (Gillespie-Lynch, 2019; Harris, 2016). A simple way to define autism is via its diagnostic criteria, defining ‘autistic people’ as those who have received an autism diagnosis. This most commonly manifests itself as the *biomedical approach* to understanding autism, where autism is considered to reflect an underlying biological and pathological difference (Chapman, 2020; Evans, 2013).

Autism was first described by Kanner (1943, p250) as an “innate inability to form the usual, biologically provided affective contact with people”, generally depicting children with severe language difficulties. This was considered a distinct neurodevelopmental disorder, and thus diagnostically excluded children with known genetic syndromes, intellectual disability (ID), or neurological injury. For decades, Kanner’s autism was considered a rare (0.02–0.04%) form of childhood schizophrenia (Harris, 2016), until its introduction to the Diagnostic and Statistical Manual of Mental Disorders third ed. (DSM-III; American Psychiatric Association [APA], 1980) as the distinct condition ‘infantile autism’. Following revisions in the DSM-IIIR (APA, 1987) and the DSM-IV (APA, 1994), the diagnostic criteria broadened to adapt to increasingly documented heterogeneity. Related disorders such as Asperger’s syndrome (Asperger & Frith, 1991) and ‘pervasive developmental disorder – not otherwise specified’ (Towbin, 2005) were assimilated as autistic subcategories. Autism then described deficits beyond poor verbal ability, such as less severe social difficulties and broader cognitive differences. Ultimately, these subcategories were deemed too unreliable, with unclear diagnostic boundaries and poor independent predictive power (Grzadzinski et al., 2013; Szatmari et al., 2009), thus they are now encapsulated by the umbrella category ‘autism spectrum disorder’ (ASD) in the DSM-5 (APA, 2013). Similar diagnostic changes occurred in parallel across editions of the International Classification of Diseases (ICD; Harris, 2016). Consequently, the diagnostic prevalence of autism has risen dramatically over the last few decades (Matson & Kozlowski, 2011), with a review of recent international studies reporting estimates between 1.48–3.62% (Fombonne, 2018). Furthermore, these recategorisations may be causing diagnostic substitutions towards autism. For example, between 2000 and 2010 in the USA, ASD diagnoses increased by 331%, whilst ID, emotional disturbance, and specific learning disability diagnoses decreased by 31%, 22% and 19% respectively, despite the proportion of total diagnoses remaining unchanged (Polyak et al., 2015). As ASD represents such breadth, some now argue it has become overinclusive and overly diagnosed (Navon & Eyal, 2016; Waterhouse et al., 2016).

Nonetheless, the current DSM-5 criteria embrace this breadth, defining ASD as a spectrum of various traits rather than a continuum of severity (Harris, 2016). Diagnosis requires meeting two key criteria, currently or by history (APA, 2013):

• “Persistent deficits in social communication and social interaction across multiple contexts”, including deficits in social-emotional reciprocity, nonverbal communication, developing, maintaining, and understanding relationships, or similar.
• “Restricted, repetitive patterns of behaviour, interests, or activities”, requiring at least two of four of (a) stereotyped or repetitive motor movements, use of objects, or speech; (b) insistence on sameness, inflexible adherence to routines, or ritualised patterns of verbal or nonverbal behaviour; (c) highly restricted, fixated interests that are abnormal in intensity of focus; or (d) hyper- or hyporeactivity to sensory input or unusual interests in sensory aspects of the environment.

Furthermore, symptoms must (a) be present from early development; (b) cause clinically significant impairment in social, occupational, or other important areas of functioning, and (c) not be better explained by ID or global developmental delay, although co-occurrence is permitted when social communication is still below expected levels. Diagnosis also clarifies degree of disorder as ‘mild’, ‘moderate’ or ‘severe’, depending on degree of impairment.

It should be apparent from the diagnostic criteria that two people might share an ASD diagnosis, but present their autistic traits in strikingly different ways. For example, their deficits in social communication and interaction (SCI) may manifest in different contexts, they may display opposite pairs of restrictive, repetitive behaviours (RRBs), and different domains of function may be impaired. Inevitably then, substantial heterogeneity exists between autistic individuals, and many argue that ASD’s diagnostic criterion are arbitrarily drawn (Müller & Amaral, 2017; Waterhouse et al., 2016). ASD’s validity as an umbrella category therefore depends on its rationale for whom it does and does not include. As its DSM-5 criteria are behaviourally defined, this rationale must be grounded in behavioural data.

ASD would have a fair rationale for its diagnostic criteria if a substantial relationship between the core diagnostic features of impaired SCI and the four RRBs existed uniquely within ASD. This would not only validate autism as a distinct group, but suggest the existence of a unified cause. A review of 36 studies using factor analysis of symptoms in autistic samples reported that most confirm two independent factors, social impairment and RRBs (Shuster et al., 2014), although some report a third by distinguishing between forming relationships and social communication. Importantly however, relationships between factors were mixed, with some studies finding strong correlations (e.g., Frazier et al., 2014; Lam et al., 2008), some weak (e.g., Happé & Ronald, 2008), and others reporting no correlation whatsoever (e.g., Harrop et al., 2014; Hus et al., 2007). Problematically, few studies considered non-ASD samples, thus overlook impairments in SCI and RRBs existing outside of autism. Those that did derived the same symptom factors in non-autistic groups, but with similar relationships and replicability issues (Constantino & Charman, 2016; Happé et al., 2006; Posserud et al., 2013). In each case then, impaired SCI does not reliably coincide with RRBs, nor reliably predict which RRBs are displayed, and vice versa.

The inconsistencies in findings likely reflects the heterogeneity both within and between samples (Levy, 2019), even with several studies achieving large 5-figure sample sizes, as participants varied greatly in ages and expression of autism. Alternately, it may expose the subjectivity of many ‘autistic traits’, which were measured using different tools such as self-report, parental report or direct observation (Shuster et al., 2014). These methods are often deemed imprecise due to vulnerabilities to biases and poor test-retest reliabilities (Chan, 2010). Jaswal & Akhtar (2019) even argue that degree of social impairment cannot be empirically characterised as ‘autistic’ or ‘non-autistic’, as social interaction is shaped within diverse cultural contexts. Therefore, although prevalence of individual symptoms is certainly higher in autistic groups, the lack of replicable correlations between them in both ASD and non-ASD samples provides only mediocre evidence that symptoms are uniquely interrelated in ASD.

ASD should also be clearly differentiable from both typical development and other disorders to be a meaningful diagnostic category. A key problem here is that both impaired SCI and RRBs exist outside of autism. For example, many TD children display similar rates of RRBs, which generally disappear with age (Camarata, 2014; Harrop et al., 2014). This may interfere with early ASD diagnosis, especially given the imprecision of symptom measurement tools. Impaired SCI is rarer in TD children, thus may be a better early indicator of autism, but without considering RRBs there is no diagnostic distinction between ASD and the similar ‘social communication disorder’ (Norbury, 2014), or perhaps even an introverted TD child (Jaswal & Akhtar, 2019). Indeed, many children display impaired SCI and only one RRB (Happé et al., 2006), further confusing these diagnostic boundaries. This problem is exacerbated by the lack of a strong evidence-based rationale for ASD’s diagnostic insistence on impaired SCI and two RRBs simultaneously (Smith et al., 2015).

There is also a lack of pure-case autism, as fewer than 5% of autistic individuals express impaired SCI and minimum two RRBs without any notable non-ASD symptoms or comorbid disorders (Lundström et al., 2015). This is not due to chance co-occurrence, rather, an ASD diagnosis is a significant risk factor for other conditions. For example, estimated prevalence for attention deficit hyperactivity disorder (ADHD) is markedly higher in the autistic versus non-autistic population, at 59% versus 5% (Polanczyk et al., 2007; Stevens et al., 2016). Similarly elevated prevalence are seen in anxiety disorders (40% vs. 18%; Zaboski & Storch, 2018), depression (23% vs. 7%; Hollocks et al., 2019), ID (50–70% vs. 1–3%; Srivastava & Schwartz, 2014), gastrointestinal problems (46–84% vs. 11–33%; Al-Beltagi, 2021) and many others (see Mannion & Leader, 2013 for review). This even includes disorders with specific genetic aetiologies, for example 61% of Rett syndrome, 16% of Down’s syndrome, and 22% of fragile X syndrome cases show comorbidity with ASD (Richards et al., 2015). Overall, it is estimated that 70% of autistic individuals show co-occurrence with at least one other neurodevelopmental or psychiatric disorder, with 40% co-occurrence for two or more (Simonoff et al., 2008). Not only then is there substantial heterogeneity within ASD, but substantial overlap with other disorders. The simplest theoretical explanation, is that each ‘autistic trait’ is each linked to different environmental, cognitive, neurobiological or genetic risk factors, but these are also risk factors for many non-ASD traits/conditions (Brunsdon & Happé, 2014). Consequently, correlations between these traits are apparent, but they are arguably too variable to warrant distinct categorisation of ASD (Müller & Amaral, 2017). Although ASD defines itself as an umbrella category, accepting heterogeneity within it, the boundaries drawn with other disorders, and oftentimes typical development, are poorly justified and ultimately arbitrary.

### Neuropsychological basis for autism as a biomedical construct

Thus far, ASD as a diagnostic group has only been considered from a behavioural perspective, through which it is deemed to have poor construct validity. Nonetheless, ASD’s biomedical usefulness may be salvaged if it has unique genetic, neurobiological, or cognitive traits, through which autism-specific explanatory theories and medical interventions may be developed.

Certainly, ASD is largely heritable. The largest aetiological study to date, spanning five countries and over 2,000,000 individuals, estimated international heritability of ASD at between 73.2–85.5% (Bai et al., 2019). This is consistent with an earlier twin study meta-analysis (64–91%, Tick et al., 2016) and other population based studies (66%; Pettersson et al., 2019; 83%; Sandin et al., 2017; 80%; Yip et al., 2018). Interestingly, heritability estimates within individual countries ranged between 50% (Finland) to 87% (Israel), which was concluded to reflect international differences in diagnostic thresholds and therefore prevalence, despite all nations using DSM-5 criteria. Heritability alone however, does not necessitate the existence of biomedical pathology. For example, personality traits such as extraversion and neuroticism show substantial heritability (approximately 40%; Vukasović & Bratko, 2015), but these are not considered to reflect distinct biological groups. Autism-specific genes and causal relationships should be identified instead. Reviews of genome-wide association studies (GWAS) conclude that whilst hundreds of genes and gene mutations have been loosely associated with ASD (Berg & Geschwind, 2012; Richards et al., 2015), only two genome-wide significant loci have been found, which are not even compellingly replicable (Anney et al., 2012). Furthermore, the majority of genes/mutations associated with ASD have been similarly associated with other disorders (Brunsdon & Happé, 2014), thus are not specific to ASD. It is likely therefore, that genetic correlates of autism represent an array of risk factors for both ASD and related non-ASD traits, rather than causes of ASD uniquely.

Similarly, many relationships between ASD and particular brain structures/networks have been found. At the group level, Riddle et al., (2017) found that 443 ASD individuals had a 2% increase in grey matter volume compared to 390 TD individuals. This finding is tenuous however, as there was substantial grey matter variation in each group, and the effect was non-significant once participants were IQ-matched. Reduced corpus callosum has also been reported (Booth et al., 2011; Paul et al., 2014), but this does not replicate with larger samples (Lefebvre et al., 2015). Macrocephaly in ASD has been has been reported by some studies (Gillberg & de Souza, 2002; Lainhart, 2015), whilst other report microencephaly (Gillberg & de Souza, 2002; Nebel et al., 2015), and some report typical head circumference (Lenroot & Yeung, 2013). This inconsistent evidence for autistic brain volume is problematic for the most popular brain impairment theory for ASD, that early brain overgrowth leads to underconnectivity (Anderson, 2013; Ecker et al., 2015). Underconnectivity is often reported in ASD, albeit in different networks, such as temporal and parietal language and “social-person” networks (Venkataraman et al., 2015), and frontal lobe-linked connections (Kitzbichler et al., 2015). Equally however, other studies find no evidence of malconnectivity, despite employing similar imaging and sampling methods (Kirkovski et al., 2015; Lefebvre et al., 2015; Redcay et al., 2013; Tyszka et al., 2014). Again, these relationships are rarely specific to ASD, with many found in related disorders such as epilepsy (Bozzi et al., 2018) or ADHD (Kasparek et al., 2015). Severely inconsistent and oftentimes contradictory evidence therefore limits neurobiological theories of ASD, once more reflecting the heterogeneity between autistic individuals and samples. Unless this replication crisis is resolved, no unified or unique neurobiological features of ASD may be discovered.

Although no unitary genetic or neurobiological features of ASD have been compellingly established, simultaneously considering multiple of the above factors may yet allow identification of a distinct autistic group. Attempts at early autism screening based on multiple genetic markers may have moderate reliability. For example, Glatt et al. (2012) successfully identified ASD in 22 of 30 autistic participants, and non-ASD in 23 of 34 controls, thus an accuracy of approximately 70%. Similarly, Dekhil et al. (2020) could successfully group approximately 80% of 561 ASD and 521 TD adolescents/adults using functional and structural magnetic resonance imaging (MRI) alone. A review of alike studies reported similar successes (Khodatars et al., 2021). Such studies however, offer only ASD or non-ASD as diagnostic choices, thus neglect the substantial diagnostic, genetic, and neurobiological overlap between ASD and other disorders. Furthermore, they each included similar proportions of ASD and non-ASD participants, despite the global base-rate of ASD being between 1.48–3.62% (Fombonne, 2018). Consequently, a fictional diagnostic tool could have a superior accuracy of >96% if it simply diagnosed everyone as non-autistic. Henceforth, current genetic and neurobiological methods of identifying ASD are not sufficiently sensitive or specific to be used in clinical practise (Varcin & Nelson, 2016), or justify ASD as a distinct biological group.

Unique or unitary cognitive traits therefore, are the final route through which ASD might be validated as a distinct group. A specific core-deficit in *theory of mind* (ToM), the understanding that individuals have unique beliefs and emotions, is the most prevalent causal explanatory theory of autism (Baron-Cohen, 2000). Poor performance in experimental ToM tasks, such as the Sally-Anne task (Baron-Cohen et al., 1985) is commonly reported in autistic samples at the group level. Often however, results do not replicate (e.g., Buitelaar et al., 1999; Ozonoff et al., 1991). ToM deficits are not specific to ASD, as other disorders such as specific leaning disabilities (Loukusa et al., 2014), and even TD children with fewer siblings (Peterson, 2000), will perform similarly by group-average (Gernsbacher & Yergeau, 2019). Furthermore, 20% of autistic children (Frith & Happé, 1994), and perhaps more (Gernsbacher & Yergeau, 2019), perform well in ToM tasks, thus impaired ToM cannot provide universal explanation for autism. It also cannot explain RRBs, or why they might coincide with impaired SCI. Other cognitive theories of autism, such as reduced social motivation (Jaswal & Akhtar, 2019), weak central coherence (Happé, 1996), and executive dysfunction (Hill, 2004), have suggested alternate universal cognitive traits in autism. Ultimately however, they too fail to offer replicable, autism-specific evidence which captures the heterogeneity within ASD (Waterhouse et al., 2016). Presently therefore, no cognitive phenomenon has been identified which justifies ASD as a distinct diagnostic group.

To summarise the above discussion, there is currently no compelling behavioural, genetic, neurobiological or cognitive evidence which supports the existence of ASD as a unique diagnostic group. Even after reimagining autism as a ‘spectrum’, the label is too broad to characterise universal traits, and diagnostic boundaries are too fuzzy to establish unique traits. Consequently, the medical aim of developing autism-specific support has been unfruitful (discussed in a later section), and consistent developmental prognoses have not been drawn. Increasingly therefore, ASD is considered to lack utility as a biomedical category (Müller & Amaral, 2017; Waterhouse et al., 2016). There is not sufficient evidence to claim that autistic individuals have categorically ‘different brains’. Nor is there sufficient evidence to claim autism is the *cause* of an autistic person’s personality or abilities.

Of course, this may eventually change. The research domain criteria (RDoC) project is using a transdiagnostic approach to conceptualise developmental and psychiatric disorders, combining a breadth of genetic, neurobiological, physiological, cognitive and behavioural data (Cuthbert, 2015). This aims to produce a biological model of these disorders, transcending the behavioural symptom-based models of the DSM-5. This may establish a biological basis to ASD, or even reshape ‘autism’ into a new biological group/groups (Daedelow et al., 2021). Outcomes are thus far unclear, but RDoC will need immense datasets which are currently unavailable. Furthermore, given the substantial heterogeneity already established in ASD, and the potential of indefinite granularity, the project may be overly optimistic (Müller & Amaral, 2017).

The neuropsychological alternative to biomedical models is the *neurodiversity approach*, which describes that ASD is merely a manifestation of natural neurobiological variation within the population (Kapp et al., 2013). Individual ‘autistic’ and ‘non-autistic’ traits share unique but overlapping risk factors (Brunsdon & Happé, 2014), thus they correlate to create the illusion of diagnostic categories, despite not supporting a categorical distinction between ASD and non-ASD (Waterhouse & Gillberg, 2014). Neurodiversity warrants an end to group-level studies using ASD-defined samples, which produce unreliable, heterogenous data that cannot be generalised towards any autistic individual, thus has poor translational clinical application (Astle & Fletcher-Watson, 2020). Instead, research may focus on understanding and supporting the individual traits previously defined as ASD’s ‘symptoms’, unencumbered by poor biomedical categorisation.

### Autism as a social construct

‘Autism’ still has meaning outside of biomedical labelling, through social constructionism (Andrews, 2012). In this sense, autism exists as a socially constructed phenomenon, gaining meaning through language, social interaction and media representation, separate from its objective scientific reality (O’Reilly & Lester, 2017). Inevitably, this varies between individuals and social contexts, given the label’s historical shifting and sociopolitical complexity.

Social constructions of autism often resemble biomedical ideologies by retaining notions of disorder and disability. Such notions largely present autism as a negative condition, thus it is commonly associated with unfavourable stereotypes and social stigma. For example, eight out of the top 10 stereotypes which non-autistic adults associated with autism were found to be negative, such as difficult personalities and awkwardness (Wood & Freeth, 2016; see also Cage et al., 2018). Even the notion of atypicality is often perceived as problematic. A thematic analysis of online forums investigating social construction of autistic pathology identified two key themes: the concept of abnormality, and the goal of normalising autistics through therapy (O’Dell & Brownlow, 2015). Commonly then, pathologising autism leads to attitudes that autistic individuals should be treated or cured, pushing them towards typicality.

Once again, neurodiversity offers an alternative to this approach. Sociopolitically (separate from neurodiversity as a neuropsychological approach), the *neurodiversity movement* actively resists the pathologising of autism, shifting its social construction’s emphasis from ‘deficit’, to ‘difference’ (Kapp et al., 2013). In line with the social model of disability (Barnes, 2019), which attributes disability to systemic and social barriers, rather than pathological impairments, most neurodiversity proponents take a value neutral perspective on autism. Here, autistic identity is sociopolitically akin to ethnicities or sexualities (Chapman, 2020; Walker, 2021), for example white heterosexual people represent the majority in western cultures, but divergence from this should not be considered impairment. Indeed, the neurodiversity movement describes autistic people as belonging to a *neurominority* group (Walker, 2021), with respect to the natural neurocognitive diversity within humanity. Most commonly however, this notion of neurocognitive diversity is channelled by the terminology of *neurodivergent* and *neurotypical*.

The neurodiversity movement’s value neutral perspective advocates consideration of an individual’s strengths and difficulties when providing support, without the biomedical demand for characterising pathology. This reimagining has a fair rationale, as many traits associated with autism are benign, or even advantageous (Akhtar & Jaswal, 2013). For example, in between-group designs, autistic children (Joseph et al., 2009; O’Riordan et al., 2001) and adults (O’Riordan, 2004) consistently perform better than IQ/age-matched TD groups in visual search tasks (note, as in upcoming citations, ‘autistic groups’ may not be representative of ‘autistic individuals’, given methodological issues of heterogeneous data). This finding is not included in DSM-5 criteria, which merely describes deficits. Similarly, special interests, considered an RRB, often show positive elements. Interview data reports that engagement in special interests is positively related to subjective wellbeing (Grove et al., 2018), with 86% of autistic adults working or studying in an area related to their special interests, consequently showing higher intrinsic motivation and superior ability (Patten Koenig & Hough Williams, 2017). Admittedly, such data may show recruitment or report bias, and in some individuals fixated interests cause sacrifice or forgetting sleep, hygiene, and important commitments (Smerbeck, 2019), thus are problematic. Nonetheless, many cases of special interests are non-detrimental or even positive. Consider also the exceptional abilities shown by rare autistic savants, in domains such as memory (Tammet, 2007) and musical talent (Young & Nettelbeck, 1995), it would be bizarre to consider these ‘impairments’.

Furthermore, deficits in SCI, the most common feature of negative stereotypes (Wood & Freeth, 2016), may be unfairly attributed towards autistic impairment. Impairments in autism are traditionally identified by comparing autistic and TD between-group means, in randomised control trial designs. Their conclusions however, such as poor ToM in autism, are often contradicted by a rich but underappreciated data source: autistic testimony (Jaswal & Akhtar, 2019). Autistic individuals often report, or are observed to have, greater affinities and understandings towards other autistic individuals (Chown, 2014; Milton, 2012; Morrison et al., 2020), comparable to those between TD individuals. This suggests that rather than impaired SCI being attributable to autistic pathology, bidirectionally poor SCI exists between autistic and non-autistic groups. For example, autistic groups often show poor emotional face recognition towards non-autistic individuals (Harms et al., 2010), but non-autistic groups also struggle to read autistic mental states (Edey et al., 2016). Similarly, whilst autistic-TD information transfer is impaired, TD-TD and autistic-autistic information transfer are similarly effective (Crompton et al., 2020). Theoretical interpretations of this phenomenon describe the *double empathy problem*, which situates the communicative difficulties within the bi-directional nature of communication, rather than solely within an autistic communicator/receiver (Milton, 2012; Mitchell et al., 2021). As such, by shedding the notion of autistic pathology and considering autism as a neurocognitive minority (Walker, 2021), the neurodiversity movement advocates that an onus for communicative change should no longer be placed on autistic individuals.

Through social construction, autism has become a salient sociopolitical label, representing people belonging to this neurominority group (Chapman, 2020; Walker, 2021). It has enabled autistic communities, such as Autism Network International (Sinclair, 2005), through which autistic individuals may meet and socialise with likeminded individuals. In line with double empathy phenomena, autistic individuals often report feeling “at home” in such environments (Sinclair, 2010). Autistic communities have developed autism-specific vocabularies, which help describe and understand autistic experience (see National Autistic Society, 2022b). This prominently includes preference of identity first language, i.e., ‘autistic person’ rather than ‘person with autism’, such that autism is presented as an inherent part of a person’s identity (Vivanti, 2019). Some even identify as autistic without a formal diagnosis (Lister, 2021). The label is ultimately pivotal to the self-identities of many autistic individuals, who often describe that they are who they are *because* they are autistic (Botha et al., 2020). Interestingly, although autistic individuals generally advocate non-pathological accounts of autism (i.e., neurodiversity, elaborated in the next section), the vast majority still describe a clear distinction between autism and non-autism (e.g., 20 of 20 autistic participants during interview; Botha et al., 2020). Suggestions that ‘everyone is on the spectrum’ are resisted by autistic communities, as seen in media articles (e.g., Doyle, 2021), and social media (e.g., Payton, 2019). Of course, when evaluating this evidence, sampling and report biases in qualitative methodology may seem limited in explanatory power, particularly compared to controlled neuropsychological designs. Nonetheless, subjective experience of self-identifying autistic individuals is highly relevant to autism’s meaning. Even if biomedical accounts of autism are abandoned, autism’s sociopolitical reality is undeniable.

Throughout social constructions of autism, whether pathological or neurodivergent, the notion of *difference* is consistently emphasised (Akhtar & Jaswal, 2013; Hacking, 2009). Difference may be conceptualised in two ways. If ‘different’ referred to, by partial analogy, the extremes within a normal distribution of multiple traits, there would be no categorical boundary between ‘autistic’ and ‘non-autistic’, save for *n* standard deviations which in itself is mathematically arbitrary. This is representative of neuropsychological models of neurodiversity, where autism manifests as the simultaneous extremes of multiple, variable, neurobiological traits (Kapp et al., 2013). Alternately, by the same analogy, ‘difference’ may refer to a bimodal distribution of traits, where a clearer boundary exists between ‘autistic’ and ‘non-autistic’ groups. This better represents biomedical models of autism, which are not supported by neurobiological evidence (Waterhouse et al., 2016). It is curious therefore, that social constructions of autism, even the neurodiversity movement, generally favour the latter (Botha et al., 2020; Chapman, 2020; Kapp et al., 2013). Autistic individuals are still perceived as having categorically ‘different brains’.

It is unclear why the notion of categorical difference is still prevalent despite poor neurobiological evidence. It is likely a combination of historical biomedical popularity (Evans, 2013), the binary nature of sociopolitical labelling (Chapman, 2020), and its heuristic simplicity (Raab & Gigerenzer, 2015). Nonetheless, the clear distinction between ‘autistic’ and ‘non-autistic’ is only made through social construction. This is not to say that it is untrue, as a key facet of social constructionism is its independence from objective scientific reality (Haslanger, 2012). It does however, indicate that it is dynamic, as socially constructed concepts may be both reconstructed and deconstructed. Autism, and the concept of ‘different brains’, may therefore be influenced by clinical and educational professionals depending on what is most beneficial for autistic individuals and society. To change how autism is socially constructed is to change what autism *is*.

## Self-Identity and Autism

The self-identity formed by autistic individuals, and the meaning they attach to the label, is central to understanding the impact of the ‘different brains’ social construction. Many autistic people report mixed feelings towards autism (Macleod et al., 2013; Mogensen & Mason, 2015), balancing feelings of self-understanding and group belonging provided by the label with experiences of difficulty and stigmatisation. These factors will therefore be discussed in turn, however the ways ‘autism’ is first introduced to autistic people, and how meaning is made of it, should first be acknowledged.

### Making meaning of one’s own autism

The meaning made of autism inevitably depends on discovery of one’s own autistic identity. This varies between individuals, largely depending on age and route of diagnosis, however individual factors such as parental wishes, emotional awareness, and support provided are also influential (Kapp, 2018). Most commonly, given the prevalence of biomedical epistemology, autism is introduced through formal diagnosis of ASD in childhood.

Early diagnosis is generally prioritised, as it is widely reported that earlier implementation of support leads to greater long-term outcomes in language and cognitive abilities (Corsello, 2005; Webb et al., 2014). A systematic review investigating age of childhood ASD diagnosis, using 56 studies across 40 countries between 2012–2019, covering 120,540 autistic individuals, reported global mean age as 60 months, with a substantial range of 30–234 months (van ’t Hof et al., 2021; see also Daniels & Mandell, 2014). Findings are mostly representative of UK studies (Brett et al., 2016; Crane et al., 2016), which report marginally older average age of diagnosis.

More challenging to ascertain however, is how diagnosis is disclosed to autistic children. Parents will usually decide when and how to tell their child about autism (see National Autistic Society, 2020c), but this varies between cases. Research in this area is scarce, with a systematic review on parental disclosure including only 34 autistic individuals (Smith et al., 2018). Many such cases reported substantial delay between diagnosis and disclosure, as parents may fear the child misunderstanding or resenting the diagnosis, or fear stigmatisation towards both autistic child and parent (Cadogan, 2015; Finnegan et al., 2014). Consequently, some autistic children have no awareness of their ASD label, estimated at 32% in an online survey of 379 parents (Crane et al., 2019). Some experience involuntary disclosure, from a non-parent relative, school, or discovering and asking about autism themselves (Riccio et al., 2021). Where disclosure occurs, immediate responses vary, including anger and denial, but also relief at an explanation for past experiences (Mogensen & Mason, 2015). Notably, autistic adolescents often retrospectively report not understanding autism until late adolescence, despite disclosure in early childhood (Jones et al., 2015), an important finding given the severe lack of research on autistic identity in young children. Commonly therefore, autistic individuals report that diagnosis meaning is self-made over time, through self-awareness of experience, peer interaction, and accessed support (van ’t Hof et al., 2021).

Quite separately, a minority of autistic individuals are diagnosed in adulthood (see Huang et al., 2020 for scoping review). Several studies indicate international prevalence of adulthood diagnoses is increasing (Bachmann et al., 2018; Diallo et al., 2018; Jensen et al., 2014), attributed to increased autism awareness and broadened diagnostic criteria. Most adults attain diagnoses through seeking social or mental health support (Geurts & Jansen, 2012), with a UK survey of 128 autistic adults reported that 44% suspected themselves to be autistic prior to diagnosis (Jones et al., 2014). Following diagnosis, many individuals report explanation for and/or confirmation of lifelong feelings of difference (Huang et al., 2020). These generally elicit positive feelings of relief, self-understanding and self-acceptance (Cooper et al., 2021; Wylie, 2014), although a minority report shock and anger (Bargiela et al., 2016). Alternately, some individuals self-identify as autistic without a formal diagnosis (Huang et al., 2020; Lister, 2021), having learned about autism through media or social interaction. These individuals usually hold positive attitudes towards autism (Sarrett, 2016), demonstrating the influence of the autism label beyond biomedical utility.

Once autistic self-identity is established, the way autistic individuals understand autism may be considered. Most autistic individuals acknowledge broad variation within autism (Macleod et al., 2013), with a minority stating that their understanding of autism comes purely from self-experience (Jones et al., 2015). It was noted earlier that autistic individuals generally advocate neurodiversity perspectives of autism, however some age differences are apparent here. In a larger scale open-ended survey including 233 autistic adults, the vast majority expressed a preference for autism as ‘difference’ rather than ‘disorder’ or disability’ (Kenny et al., 2016). Similarly, in an interview of 20 autistic adults, most described autism as value-neutral, akin to height or handedness, whilst making clear arguments for a causal biological basis (Botha et al., 2020), i.e. ‘different brains’. Furthermore, autistic adults often express pride towards the label as a fundamental aspect of their self-identity, rather than something they have (Botha et al., 2020; Cooper et al., 2021). Autistic adolescents show more mixed responses. In semi-structured interviews, few considered autism a disability when explicitly asked (Jones et al., 2015), but some did describe it in terms of difficulties it ‘causes’ (Mogensen & Mason, 2015). More positively, many autistic adolescents described autism as a validating explanation for their unique behaviours and feelings of difference (Huws & Jones, 2009; Jones et al., 2015; Mogensen & Mason, 2015), again with references to the brain. Interestingly, although some autistic adolescents referenced themselves when describing autism, very few mentioned autism when asked to describe themselves (DeNigris et al., 2018; Riccio et al., 2021), suggesting the label may be less salient to the self-identities of younger autistic people.

Some attempts have been made to predict perceptions of peoples’ own autism. Findings that autistic adolescents tend to describe autism less favourably than adults have induced suggestions that greater experience and understanding of autism enhances attitude (Berkovits et al., 2020; Drummond, 2013). This is especially apparent in individuals diagnosed in adulthood, who likely experienced autism-related difficulties for longer, and/or independently learned about autism, before accessing the support and validation provided by the label (Cooper et al., 2021; Cresswell & Cage, 2019). Consequently, it is likely that greater emphasis and teaching about autism at younger ages may accelerate these positive attitudes. Indeed, the role of parental disclosure and discussion seems highly influential. In cases where little or no parent-child discussion about autism had occurred, weak association between parent and child descriptions of autism were found in an interview of 19 adolescent-parent pairs, despite similarities at the group level (Riccio et al., 2021). Similar analyses report that autistic adolescents who experienced voluntary parental disclosure commonly view autism more positively, and use more neurodiversity aligned language (Humphrey & Lewis, 2008; Riccio et al., 2021; Russell & Norwich, 2012). Active parental discussion, emphasising difference over deficit, is therefore encouraged. It should be noted however, that these trend’s effect sizes appear modest, and predominantly qualitative and exclusively cross-sectional data limits statistical and longitudinal testing (Han et al., 2022). Other factors have been associated with facilitating positive autism understanding, such as emotional awareness of difference prior to diagnosis, and neurodiversity aligned educational/social support (Kapp, 2018), however evidence is mostly limited to qualitative reports. As meanings attributed towards autism are learned through complex interaction of social factors, a large proportion of their variance remains unexplained.

### Autism as social identity

As a social construction, autism has become a salient social identity, referenced by many autistic individuals as central to their self-identity. Indeed, there is consensus that the most common feature of positive self-identities is the validation provided by being recognised and accepted as part of an autistic group (Han et al., 2022). This may be understood through social identity theory (SIT; Tajfel & Turner, 2004), which proposes that people naturally form social groups, and define themselves by these group memberships. Consequently, group memberships provide individuals with feelings of well-being and self-esteem, given perceived shared characteristics and achievements of the group (Jetten et al., 2012). Evidence for, and applications of SIT are well documented amongst a variety of contexts (Hogg, 2016), and it may explain common beliefs of categorical difference between autism and non-autism (Botha et al., 2020), constructed by in-group out-group dynamics. Indeed, evidence for autism as a social identity is substantial. In qualitative research, both autistic adolescents (Jones et al., 2015; Mogensen & Mason, 2015) and adults (Botha et al., 2020; Cooper et al., 2021) often describe a sense of belonging within the autistic community, or report the achievements of other autistic individuals with pride. For many autistic individuals, reclamation of self-identity through group membership, by independently researching autism and meeting other autistic individuals, strengthens feelings of self-understanding and self-acceptance (Han et al., 2022).

Importantly, quantitative data exists to supports these claims, with substantial effect sizes. In a survey of 272 autistic adults (Cooper et al., 2017), regression analyses indicated that degree of autistic self-identification significantly predicted perceived positivity of autistic identity (considering both the in-group and out-group’s attitude towards autism, coined *collective self-esteem*), which in turn predicted personal self-esteem (see also Cooper et al., 2021). Personal self-esteem subsequently predicted lower anxiety and depression ratings. Similarly, through surveying 111 autistic adults, higher autistic self-acceptance significantly predicted lower depression ratings, whilst controlling for age, gender, and age of diagnosis (Cage et al., 2018). Importantly, the present research could only find compelling quantitative data for autistic adults, leaving the possibility that such trends do not exist in autistic adolescents. Indeed, a similar but rare analysis on autistic adolescents found no significant differences in mental health between autistic and non-autistic aligned individuals, although samples were too small to achieve meaningful statistical power (*n* = 3 and 7 respectively; Cresswell & Cage, 2019). Nonetheless, positive social identification as autistic is clearly associated with better self-esteem and mental health in autistic adult populations, and although cross-sectional quantitative data cannot demonstrate causality, qualitative reports consistently describe a causal link.

As a social identity however, autism may become subject to stereotyping and stigma. Indeed, the most common theme when describing negative attitudes towards autism is the feeling that autism, both label and behaviour, is stigmatised by the neurotypical population (Han et al., 2022). Experimental research on SIT suggests that membership of a stigmatised group may still be perceived positively (Crocker & Major, 1989), provided the individual develops high collective self-esteem (Cooper et al., 2017). In such cases, personal self-esteem is somewhat protected from discrimination and negative feedback by these being attributed towards prejudice against the group. Indeed, many autistic individuals describe this effect when discussing the self-acceptance provided by the label (Botha et al., 2020; Han et al., 2022). Where individuals reject or attempt to conceal their autistic identity however, aversive social experiences may have more damaging effects.

For many autistic individuals, these negative social experiences are common. In qualitative research, both adolescent and adult autistic groups regularly report experiences of being insulted or socially excluded (Berkovits et al., 2020; Humphrey & Lewis, 2008). A systematic review spanning 5625 autistic children/adolescents aged 6–21 estimated that 44% experience school bullying, far higher than TD peers (Maïano et al., 2016), and often persisting in adulthood (Umagami et al., 2022). Consequently, autistic individuals sometimes report feeling marked by the label (Jones et al., 2015), and perceived as “weird” by peers (Treweek et al., 2019). Many describe that neurotypical people’s perceptions of autism focus too heavily on deficiency and negative attributes (Mogensen & Mason, 2015), which is felt to gradually propagate stigma (Linton, 2014). This leads to anticipation of stigma being reported by many autistic individuals (Johnson & Joshi, 2016), who often feel the need to conceal or camouflage their autism to appear neurotypical (see Han et al., 2022 for a systematic review of coping strategies). Camouflaging, which is attempting to consciously or unconsciously mirror neurotypical behaviour, has mixed successes in reducing stigma, although it is usually described as tiring and effortful, and reinforcing of beliefs that autism is problematic (Schneid & Raz, 2020). Henceforth, many individuals report selective and strategic disclosure of their autistic identity in different circumstances, depending on whether they expect to be accepted or further excluded (Leven, 2020; Saunders, 2017). Often however, autistic individuals describe a ‘double bind’ (Botha et al., 2020), where both disclosure and concealment of the label result in negative outcomes (Thompson-Hodgetts et al., 2020).

Unsurprisingly, quantitative data has consistently associated these aversive experiences with poorer mental health and quality of life (QoL) in autistic populations (Han et al., 2022). For example, survey data from 1139 autistic adults found that perceived stigma was associated with lower self-esteem and QoL (Mcdonald, 2016). Similar findings were made by surveying 223 autistic adults (Perry et al., 2022), which additionally associated perceived stigma with self-reported camouflaging. Notably, camouflaging did not mediate the relationship between stigma and mental health, suggesting it is an ineffective coping strategy. These findings have been replicated in 111 autistic adults (Botha & Frost, 2020), whilst controlling for general life stress and demographic factors. Follow up investigation found that higher levels of disclosure at baseline predicted better mental health 9 months later, providing rare longitudinal evidence of the positive impacts of autism disclosure (Botha, 2020). Once more, causality is described by qualitative data, with many autistic individuals describing autism as an invitation for stigma (Han et al., 2022).

External stigmatisation may even cause self-stigma in autistic individuals, where negative stereotypes are internalised (Botha & Frost, 2018). Such individuals have described themselves as having a “bad brain” (Humphrey & Lewis, 2008) or being “broken” (Leedham et al., 2020), resenting their own autism. Beyond detriment to perceived self-worth and mental health, such attitudes may also effect self-determination, given a lack of perceived competence (see Deci & Ryan, 2012 for self-determination theory). Indeed, some autistic adolescents report using autism as an excuse for their perceived lack of self-control regarding temper tantrums (Mogensen & Mason, 2015). Few quantitative studies have estimated the prevalence of self-stigma, although fortunately, it appears to be a minority. One survey of 45 autistic adults found moderate/high self-stigma in 22% (Dubreucq et al., 2020), and another reported it in 15% of 149 autistic adults (Bachmann et al., 2019), with sample characteristics suggesting moderate generalisability (Han et al., 2022). Nonetheless, inviting protective positive autistic group-membership, and reducing external stigmatisation, should be priorities to protect these individuals.

To my knowledge, no quantitative study has explicitly investigated the relationship between extent of autistic self-identity, collective self-esteem, experiences of stigmatisation, and personal self-esteem and mental health. However, through cautiously collating available evidence, it is apparent that positive autistic social identity may offer substantial protection against stigmatisation’s aversive affects (Han et al., 2022). It is fortunate therefore, that the majority of individuals in both qualitative and quantitative samples perceive autistic identity positively, thus benefiting from this protective phenomenon (Bachmann et al., 2019; Botha et al., 2020; Cooper et al., 2021; Dubreucq et al., 2020; Han et al., 2022). Consequently, factors which promote these positive attitudes, such as active parental discussion and experience of autism (Kapp, 2018), are encouraged. Crucially, the attribution for feelings of difference are often characterised by descriptions of ‘different brains’ in autistic testimony (Botha et al., 2020; Mogensen & Mason, 2015). This may also provide the unification through which the social group is defined. In this sense, present social constructions discussing ‘different brains’ may facilitate the self-acceptance and social identity provided by the label. Without compelling evidence that ‘different brains’ also causes harm, attempting to change this social construction of autism may unnecessarily undermine this positive influence of the label.

The substantial harms caused by autistic stigma may supply this need for change. However, given the atypical behaviour associated with autism, and autistic descriptions of a ‘double bind’, some argue autistic individuals would likely feel different and experience unfavourable judgement regardless of labelling (Davidson & Henderson, 2010). In such case, the label’s positive influence may exist independently from autism’s stigmatisation. To fully evaluate the social impact of labelling therefore, the object of stigma must be understood, thus this will be discussed next.

## Stigma: Autism Label or Autistic Behaviour?

Autistic people often report stigmatisation, defined as stereotyping and discrimination from a more powerful group (Link & Phelan, 2003), as the greatest difficulty they experience (Han et al., 2022). It creates substantial barriers, as many autistic individuals struggle to form and maintain social and romantic relationships with neurotypical individuals, despite expressing desires to do so (Cresswell & Cage, 2019). Equally, higher rates of bullying (Maïano et al., 2016) and difficulties securing employment compared to IQ-matched TD groups (Taylor et al., 2015; Taylor & Seltzer, 2011) have been attributed to unfavourable judgements towards autistic individuals. Families and caregivers of autistic people also regularly report experiences of both external and internalised stigma (Mitter et al., 2019). Promoting acceptance of autistic individuals is therefore essential, for which the cause of aversive social judgements must be understood. Autistic testimony often describes a ‘double bind’, experiencing stigma whether the label is disclosed or concealed (Botha et al., 2020), thus this should be investigated from the perspective of the non-autistic population. Where stigmatisation occurs specifically towards the label, autistic social identity becomes double-edged, eliciting negative stereotyping and possibly out-group derogation (see Hewstone et al., 2003). Solutions would require improvement of autism-specific attitudes, or minimisation of the label. Where stigma occur towards autistic behaviour, independently from labelling, autistic social constructions cannot be explicitly blamed, and more general anti-intolerance strategies are required.

To untangle this problem, a substantial body of research has investigated the effect of labelling on judgements towards autistic individuals (for review see Thompson-Hodgetts et al., 2020). This often measures first impressions, which drastically impact job interviews, dating, and friendship formation (Harris & Garris, 2008), and are resistant to change (Ambady et al., 2000). These experimental designs provide valuable quantitative data, and exhibit excellent internal validity by using genuine autistic people as stimuli. Across three experiments comparing autistic and TD participants in short audio-visual stimuli, 349 TD observers judged autistic stimuli significantly less favourably on measures such as awkwardness, likability, and intention to interact with, when blinded to diagnostic status (Sasson et al., 2017). Effect sizes were large, were demonstrated for both adult and young adolescent observers and stimulus participants, and occurred for audio-visual, audio-only, video-only, and static image conditions. Interestingly, biases disappeared when only non-audio-visual speech content was available, suggesting communicative style, not content, is judged unfavourably. The effect was replicated with 215 TD adults, with the important additional finding that labelling as autistic, whether accurately or inaccurately, largely improved average favourability towards stimuli (Sasson & Morrison, 2019). These judgement and disclosure effects are remarkably consistent in group-level designs, regardless of age (including children), IQ or autistic traits of observers, and have also been demonstrated using written vignettes and cartoons (Engel & Sheppard, 2020; Faso et al., 2015; Grossman, 2015; Matthews et al., 2015). These paradigms provide evidence that autistic behaviour is the primary object of stigma, likely caused by double empathy discrepancies in SCI between autistic and non-autistic groups (Milton, 2012). Labelling meanwhile, offers an alternate attribution for perceived atypicality, generally improving leniency of behavioural expectations (Thompson-Hodgetts et al., 2020).

Furthermore, in studying first-impressions of 505 TD observers towards exclusively autistic stimulus participants, Morrison et al. (2019) demonstrated that observer characteristics explain more variance in judgement ratings than autistic stimuli characteristics (besides awkwardness), particularly for intention to interact. Observers exhibiting lower self-rated autistic stigma, and to a lesser extent, increased autism knowledge, showed greater increases in favourability following autism labelling. The minority of observers who heavily stigmatised autism however, showed the reverse effect, suggesting positive disclosure effects depend on pre-existing neutral/positive attitudes towards autism (see Jones et al., 2021 for complete replication). These findings are crucial, demonstrating that TD conceptualisations of autism are key for prevention of stigma.

TD people’s knowledge and attitudes of autism have been investigated by numerous large-scale studies (see Turnock et al., 2022 for review). Those with diverse demographics generally find no relationships for education, age, or gender (Turnock et al., 2022; see Tipton & Blacher, 2014 for marginally higher female acceptance). This suggests good generalisability for studies featuring disproportionately highly educated groups (such as many first-impression studies; Thompson-Hodgetts et al., 2020). TD descriptions of autism often reference the brain and neurobiological differences (Gillespie-Lynch et al., 2015), with the majority 823 TD adults plus 176 childcare workers identifying primary causes as neurological or genetic, although 10% named vaccines (Mitchell & Locke, 2015). Another survey of 1054 TD adults reported that 81.5% displayed moderate/high autism knowledge, and 81.3% had neutral/positive attitudes towards autism (Kuzminski et al., 2019). Indeed, many studies find strong relationships between knowledge and positive attitudes (Kuzminski et al., 2019; Ling et al., 2010; Obeid et al., 2015).

Problematically however, measures of autism knowledge/attitudes are often overly focused on semantic facts and explicit stigma. More in-depth analyses suggest that certain negative misconceptions, such as ‘autistic people do not show affection or attachments’ (Obeid et al., 2015), may dominate attitudes, regardless of other correct knowledge (White et al., 2019). Likewise, autism acceptance training for TD adults often improves explicit ability estimates in first-impression paradigms, (Gillespie-Lynch et al., 2015), but not implicit biases such as intention to interact and unpleasant personal judgements (Dickter et al., 2020; Jones et al., 2021). Knowledge and explicit stigma therefore, are not fully indicative of implicit autism acceptance. Rather, this is better predicted by increased contact and experience with autistic people (Gardiner & Iarocci, 2014; Kuzminski et al., 2019), which may falsify aversive preconceptions. Resisting harmful stereotypes and promoting contact and inclusion, should therefore be prioritised to reduce stigma.

Experimental evidence suggests that autistic behaviour is stigmatised independently from labelling, and predicts predominantly positive disclosure effects. As this evidence overlooks implicit biases in attitudes however, the latter finding is often refuted by autistic experience of the ‘double bind’. Consequently, more research into improving implicit attitudes in real-world contexts is warranted (Thompson-Hodgetts et al., 2020). Nonetheless, experimental evidence still demonstrates that label disclosure, generally interpreted as indicating ‘different brains’ (Gillespie-Lynch et al., 2015), may change attributions of behaviour hence improving explicit judgements. If negative stereotypes and misconceptions may be prevented, implicit biases may not interfere with these positive effects. Certainly, positive disclosure experiences are still reported within autistic testimonies (Thompson-Hodgetts et al., 2020), presumably where no negative stereotyping occurs. As such, social constructions of ‘different brains’ do not inherently elicit stigma via out-group derogation, rather, stigmatisation of the label prevails through misconceptions of autistic psychopathy.

## Supporting Autistic People

The purpose of biomedical study is to treat symptoms and difficulties. Although biomedical models of autism are unconvincing, sociopolitical emphases on diagnosis are still prevalent. It is therefore worth considering whether this biomedical social construction facilitates or encumbers autistic individuals in accessing effective support.

Achieving diagnosis is often fraught with difficulty and frustrating wait times. For example, a UK open-access survey of 1047 parents found that most (92%) childhood diagnoses required multiple referrals, with average duration between initial consultation and confirmation of diagnosis reported as 3.5 years (Crane et al., 2016). The most recent National Health Service data for 2019–2021 (NHS Digital, 2022) reports similarly problematic delays. During this period, support is difficult to access (Crane et al., 2018), as autism-specific services are not yet available. Meanwhile, mental health services such as Child and Adolescent Mental Health Services (CAMHS) sometimes refuse mental health support on the basis that ‘suspected autism’ is the root cause (Read & Schofield, 2010; recall that ASD and mental health difficulties commonly co-occur; Mannion & Leader, 2013). Autistic adults experience similar diagnostic challenges (Huang et al., 2020), and often feel unsupported following diagnosis (Jones et al., 2014), as most therapeutic/social support is given to autistic children/adolescents. Furthermore, given the poor rationale for ASD’s diagnostic boundaries (as discussed earlier), some individuals may not meet diagnostic thresholds despite experiencing similar difficulties. Given these problems, diagnosis may be an arbitrary gatekeeper to support for many autistic, and some non-autistic, individuals.

Diagnosis would have value however, if it enables autism-specific therapies or interventions. Importantly, in keeping with emphases on difference not disability, interventions which push autistic children towards neurotypical social behaviour often have damaging outcomes, thus are discouraged (Jaswal & Akhtar, 2019). Whilst many school-based support strategies become accessible with diagnosis, autism-specific psychological theory does not inherently inform these. For example, many children undergo applied behavioural analysis (ABA), a highly individualised intervention in both goals and application (Roane et al., 2016). ABA uses principles of operant conditioning (Staddon & Cerutti, 2003), shaping behaviour through reinforcement and punishment (although ethically, reinforcement is preffered; Jarmolowicz & Tetreault, 2015), therefore only utilising associative learning theory. Individualised goal behaviours may include language and social skills, self-care and hygiene, motor skills, learning skills, and others, with rewards also individualised. As goals and psychological principles are not autism-specific however, many non-autistic children with behavioural or developmental disorders also undergo ABA (Rodgers et al., 2021). Similar arguments may be made for speech and occupational therapies (Houtrow et al., 2019), given that treatment plans are individualised regardless of diagnosis.

Some smaller-scale interventions target difficulties associated with RRBs. For example, visual timetables and advanced preparation for changes in routine may relieve anxiety in autistic children displaying insistence on sameness (Murray, 2007). Teachers using clear, unambiguous language may prevent literal misinterpretation (Kalandadze et al., 2018), and opportunities for time-outs may reduce meltdown events (Ryan, 2010). Here, ASD diagnosis may raise awareness of these potential needs, although RRBs are not exclusively autism-specific (Shuster et al., 2014), and unique support is required for each autistic child. Indeed, teachers making false assumptions about the needs of autistic children can be unproductive and even patronising (Brownlow et al., 2021; Cameron, 2014). For example, autistic adolescents sometimes feel that unnecessary use of picture cards undermines their social competence (Mogensen & Mason, 2015). Ultimately therefore, ASD diagnosis alone cannot determine support required, clinical and educational professionals must understand that individualised approaches are necessary.

The greatest value of ASD labelling in supportive contexts comes through economic utility. Diagnosis enables access to disability benefits (see National Autistic Society, 2022a), and largely mediates allocation of educational support. It would be difficult to move social constructions of autism away from emphasising diagnoses, as disbanding the boundary between autistic and non-autistic would make allocation of support unclear. Furthermore, diagnosis is the primary source of autistic identity, which often has positive well-being and social outcomes (as discussed earlier). For these reasons, social constructionist educational approaches which minimise importance of diagnosing pathologies (Wagner, 2000) are scarcely used by educational psychologists (Farrell & Woods, 2015), despite showing promising educational outcomes where applied (Kennedy et al., 2010; Wagner, 2008).

Although ASD diagnoses may arbitrarily gatekeep support for many, solutions to this problem are likely beyond the scope of autism’s social construction. Rather, a societal paradigm shift is needed for fairer and more productive allocation of support, with lesser emphasis on biomedical diagnoses (Ho, 2004). For the near-future, economic limitations likely render this proposition idealistic. Henceforth, social constructions of ‘different brains’ are convenient for providing autism support largely through economic utility.

## Should We Continue to Tell Autistic People that their Brains Are Different?

The notion that ‘autistic brains are different’ is under intense neuropsychological scrutiny. Current evidence cannot convincingly define autism, let alone identify any categorical or causal neuropsychological differences between autism and non-autism (Happé et al., 2006; Waterhouse et al., 2016). Changes in biomedical and scientific conceptualisations of autism are clearly required, with suggestions ranging from emphasising that ‘ASD’ is a poorly generalisable umbrella term (Müller & Amaral, 2017), to full-scale disbanding of autism labels in research and practise (Waterhouse & Gillberg, 2014).

Despite this, notions of categorical, neuropsychological difference are key to autism’s social construction. Provided these are perceived as neurodivergent differences rather than pathological disability, autistic identity may have remarkably positive effects. As a social identity, autism enables feelings of positive well-being, self-acceptance, and pride to many (Han et al., 2022). Likewise, offering perceived explanation for the difficulties associated with autism allows these to be reframed as neurodivergent differences. This improves self-esteem and self-understanding in many autistic individuals (Kapp et al., 2013), and potentially reduces the social stigma they experience (Thompson-Hodgetts et al., 2020). A review by Kapp (2018) concluded that social relationships and subjective well-being predict autistic people’s self-rated QoL far more strongly than severity of autistic ‘symptoms’. Consequently, attempting to dismantle social constructions of ‘different brains’, to further neurodevelopmental understanding and translational support, may be counterproductive for supporting these individuals.

Within present social constructions of autism, two key problems are apparent. The first is the stigmatisation towards the autism label, including minority cases of self-stigma, with unfavourable stereotyping and misconceptions causing negative implicit attitudes (Jones et al., 2021). Consequently, the scientific community should continue to support the neurodiversity movement in promoting autism acceptance, beyond mere awareness, emphasising that ‘different brains’ does not necessitate deficits in empathy and social motivation (Jaswal & Akhtar, 2019). Equally, promoting interpersonal contact and inclusion (Kuzminski et al., 2019), and understanding of autistic heterogeneity, may reduce generalisations of stereotypes propagated by autism’s narrow depiction in current media (Draaisma, 2009). Constructive autism discussion should also be encouraged in parents and peers of autistic children, to accelerate positive autistic self-identity.

Secondly, biomedical demand for ASD diagnosis makes accessing support challenging. Although it is a useful gateway to autistic identity, achieving diagnosis is frustratingly inefficient (Crane et al., 2016), and subsequent support is not sufficiently autism-specific to justify this requirement. Meanwhile, where clinicians and educational professionals attempt blanket autism treatment (Brownlow et al., 2021), or deny access to mental health/other support services by blaming autism as the root cause (Read & Schofield, 2010), biomedical use of the autism label becomes explicitly counterproductive. Henceforth, awareness of autism’s limited neuropsychological validity should be encouraged within clinical and educational communities. If awareness of this problem increases, it is difficult to predict how ASD’s poor biomedical utility will interact with economic factors in determining the future of diagnostic practise. Indeed, changes in diagnostic practise would likely affect autism’s socially constructed meaning. This is largely beyond the scope of the present discussion, which merely argues in defence of the current lay social constructions of autism, but it posits an interesting question for future literature. Given autism’s dynamic history (Harris, 2016), future changes seem almost inevitable.

There are several limitations in the current evidence-base which may bias present conclusions. Most importantly, younger autistic children are widely unresearched, particularly regarding autistic self-identity. Whilst it is accepted that interview biases (see Christensen & James, 2008) makes qualitatively researching children challenging, potentially exacerbated by impaired SCI in autism, understanding the label’s short-term influence on children’s self-identity is paramount. Although retrospective reports suggest limited effects (Jones et al., 2015), recall bias is well documented in retrospective data (Coughlin, 1990), thus these findings must be interpreted cautiously. Similarly, substantial discrepancies between parent-report and adolescent self-report (Hong et al., 2016; Riccio et al., 2021), with parents more likely to hold biomedical perspectives and focus on difficulties, suggest that other-report may be an unreliable solution. Until this challenge is resolved, this gap in understanding is problematic. Furthermore, research often uses disproportionately verbally able autistic samples (hence overrepresenting autism in mainstream education), predominantly due to methodological difficulties in interviewing non-verbal individuals. As this represents approximately one third of autism cases (Rose et al., 2016), present conclusions may not generalise towards this demographic of the autistic population. Henceforth, the recommendations made by this review are only able to compellingly consider the outcomes of verbally able autistic individuals in later development.

Autism has been accused of being an arbitrary, unscientific “convenient fiction”, which has blocked progress in neurodevelopmental understanding (Waterhouse et al., 2017, p.1182). Yet, this quality has allowed autism to become a salient social construction, irrespective of scientific reality. As a social identity and attribution of difference, autistic identity has strikingly positive effects on the QoL of many autistic individuals, who would likely experience worsened well-being and stigma without it. Henceforth, although clinical and educational professionals must understand autism’s neuropsychological controversy themselves, they may have little to gain from imposing it onto lay social constructions of autism. To actively resist the “convenient fiction” that ‘autistic people have different brains’ would undermine the positive social reality of autistic identity.
